# Artificial intelligence versus physician decision-making in acute heart failure: a comparative evaluation of four contemporary AI systems

**DOI:** 10.1093/ehjdh/ztag169

**Published:** 2026-10-24

**Authors:** Georgios E Zakynthinos, Evangelos Oikonomou, Iason- Daniil Zakynthinos, George Dimeas, Panteleimon Pantelidis, Theodore G Papaioannou, Gerasimos Siasos

**Affiliations:** 3rd Department of Cardiology, ‘Sotiria’ Chest Diseases Hospital, Medical School, National and Kapodistrian University of Athens, Mesogeion 152, Athens 11527, Greece; 3rd Cardiology Department, ‘Sotiria’ General Hospital of Chest Diseases of Athens, Athina Ioanni Martinou Endowed Chair, Medical School, National and Kapodistrian University of Athens, Athens, Greece; School of Electrical and Computer Engineering, National Technical University of Athens, Athens, Greece; 3rd Department of Cardiology, ‘Sotiria’ Chest Diseases Hospital, Medical School, National and Kapodistrian University of Athens, Mesogeion 152, Athens 11527, Greece; 3rd Department of Cardiology, ‘Sotiria’ Chest Diseases Hospital, Medical School, National and Kapodistrian University of Athens, Mesogeion 152, Athens 11527, Greece; Department of Biomedical Engineering, Medical School, National and Kapodistrian University of Athens, Athens 11527, Greece; 3rd Department of Cardiology, ‘Sotiria’ Chest Diseases Hospital, Medical School, National and Kapodistrian University of Athens, Mesogeion 152, Athens 11527, Greece

**Keywords:** Artificial intelligence, Large language models, ChatGPT, DeepSeek, Heart failure, Clinical decision support

## Abstract

**Aims:**

Large language models (LLMs) are increasingly used by the public and healthcare professionals for clinical reasoning. However, they were developed as general-purpose language models rather than dedicated clinical decision-support tools, and their performance in real-world acute heart failure (AHF) management remains insufficiently evaluated. We compared four contemporary AI models with physician decision-making.

**Methods and results:**

This single-centre retrospective observational study incorporated a cross-sectional AI evaluation of consecutive patients treated at Sotiria General Hospital between July and November 2025, excluding acute coronary syndrome. ChatGPT, ChatGPT Plus, DeepSeek, and an ESC guideline-based retrieval-augmented generation (RAG) model independently assessed identical standardized cases. Recommendations were compared with physician decisions on diagnosis, treatment, guideline-directed medical therapy, timing, and prognosis using Cohen’s kappa, McNemar’s test, Cochran’s *Q*, Wilcoxon signed-rank testing, and multivariable logistic regression. Diagnostic performance differed significantly across systems (Cochran’s *Q* = 8.477, *P* = 0.037). In the balanced diagnostic cohort, ChatGPT Plus achieved the highest heart failure (HF) accuracy (accuracy 86.5%, sensitivity 87.0%, and specificity 86.0%), followed by RAG (accuracy 77.5%), ChatGPT (accuracy 77.0%), and DeepSeek (accuracy 69.0%). For HF within the top three differential diagnoses, sensitivity was 90.0–97.0%; specificity was 70.0% for ChatGPT Plus, 68.0% for RAG, 19.0% for DeepSeek, and 15.0% for ChatGPT. Physician agreement was highest for guideline-directed therapies, with greater variability in prognosis and escalation of care. RAG more closely matched physician therapeutic timing but did not consistently outperform general-purpose models.

**Conclusion:**

Contemporary AI models show promise in AHF decision support but substantial task-specific variability, supporting use as complements rather than replacements for physician judgement.

## Graphical Abstract

**Graphical Abstract:**
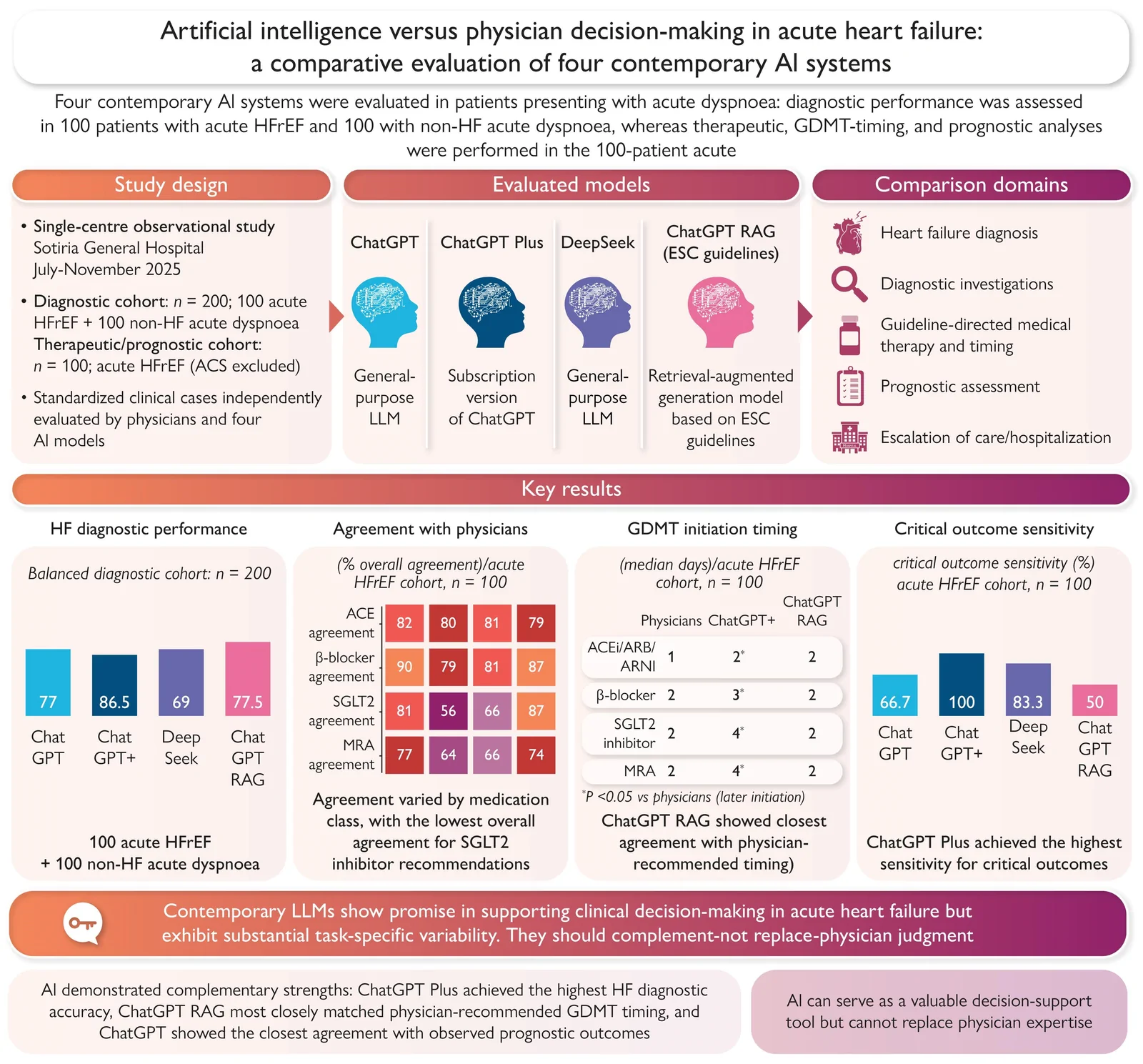
Comparison of physician decision-making with four contemporary AI systems (ChatGPT, ChatGPT Plus, DeepSeek, and an ESC guideline-based RAG system) in 100 consecutive patients presenting with acute heart failure. The models were evaluated across six clinical domains: heart failure diagnosis, diagnostic investigations, guideline-directed medical therapy (GDMT) recommendations, timing of GDMT initiation, prognostic assessment, and escalation of care/hospitalization. Key findings demonstrated complementary strengths among the models: ChatGPT Plus most frequently identified heart failure with reduced ejection fraction (HFrEF), ChatGPT RAG most closely matched physician-recommended timing of GDMT initiation, and ChatGPT Plus achieved the highest sensitivity for detecting critical outcomes. Overall, contemporary AI systems show promise as clinical decision-support tools but exhibit substantial task-specific variability and should complement—not replace—physician judgement. ACS, acute coronary syndrome; ESC, European Society of Cardiology; GDMT, guideline-directed medical therapy; HFrEF, heart failure with reduced ejection fraction; LLM, large language model; MRA, mineralocorticoid receptor antagonist; RAG, retrieval-augmented generation; SGLT2, sodium-glucose cotransporter-2.

## Introduction

Artificial intelligence (AI), particularly large language models (LLMs), has rapidly become integrated into everyday life. Beyond their widespread use by the general population, these systems are increasingly being adopted by healthcare professionals to obtain medical information, summarize scientific literature, assist with clinical reasoning, and support educational activities.^1^ As their accessibility and capabilities continue to improve, LLMs are progressively becoming part of routine medical practice.^1,2^

Despite this growing adoption, the true clinical utility of these models remains uncertain. Most currently available LLMs were developed as general-purpose language models rather than dedicated clinical decision-support systems and were not specifically designed to manage real-world patients.^1^ Consequently, although they have demonstrated impressive performance in medical examinations and various knowledge-based tasks, relatively few studies have evaluated their ability to support physicians throughout the complete diagnostic and therapeutic management of actual clinical cases. Furthermore, comparisons between multiple contemporary AI models and routine physician decision-making remain limited.^1,3^

Heart failure (HF) represents an ideal clinical setting in which to evaluate these systems. It is one of the leading causes of emergency department visits and hospitalizations worldwide and remains associated with substantial morbidity, mortality, and healthcare expenditure despite major therapeutic advances.^4,5^ The management of patients presenting with acute heart failure (AHF) requires a sequence of complex clinical decisions, including differential diagnosis, selection of appropriate investigations, determination of the optimal level of care, estimation of prognosis, initiation of guideline-directed medical therapy (GDMT), and planning of follow-up.^6^ These decisions are performed daily by cardiologists and emergency physicians and therefore provide an appropriate framework for assessing AI clinical reasoning using standardized retrospective representations of real patient cases.^6^

Recent developments have also introduced retrieval-augmented generation (RAG) systems, which combine the reasoning capabilities of LLMs with retrieval of information from curated medical resources such as clinical practice guidelines.^7^ In theory, this approach may improve guideline adherence while reducing unsupported or outdated recommendations. However, evidence comparing guideline-based RAG systems with conventional LLMs in everyday clinical practice remains scarce.^7^ Because commercial AI systems are updated rapidly, evaluations of their clinical performance should be interpreted as time-stamped assessments of specific model versions rather than as permanent estimates of performance.

Therefore, the aim of the present study was to compare the performance of four contemporary AI systems—ChatGPT, ChatGPT Plus (ChatGPT+), DeepSeek, and an ESC guideline-based RAG model—with physician decision-making in consecutive patients presenting with AHF. We evaluated the ability to identify HF, recommendations for therapeutic management, timing of GDMT, prognostic assessment, and hospitalization planning in order to determine the current clinical capabilities and limitations of these AI systems using standardized clinical representations derived from routine cardiology practice.

## Methods

### Study design and patient population

This single-centre retrospective observational study included two retrospective patient cohorts comprising a total of 200 patients and incorporated a cross-sectional evaluation of AI-system performance at a predefined time point. The therapeutic evaluation cohort consisted of 100 consecutive patients with a final diagnosis of acute HFrEF, defined according to ESC criteria as acute or worsening HF symptoms and signs with objective evidence of congestion or cardiac dysfunction and a left ventricular ejection fraction ≤ 40%. Patients with acute coronary syndrome, diagnosed according to ESC criteria using clinical presentation, electrocardiography, serial cardiac troponin measurements, and imaging or coronary angiography when indicated, were excluded because of their distinct diagnostic and therapeutic pathway.

To evaluate diagnostic performance, an additional comparator cohort of 100 consecutive patients presenting with acute dyspnoea due to causes other than HF was retrospectively identified. These patients were evaluated in the same clinical setting using the identical AI evaluation procedure and served as the disease-negative reference group for estimation of specificity, predictive values, and overall diagnostic accuracy. They underwent the identical AI evaluation procedure and served as the disease-negative reference group for estimation of specificity, predictive values, and overall diagnostic accuracy.

Treating physicians were blinded to the AI evaluation, which was performed retrospectively and did not influence patient management.

The final diagnosis was established by the treating physicians during routine clinical care using all available clinical information, including medical history, physical examination, laboratory findings, electrocardiography, imaging, and echocardiography where appropriate, as documented in the medical records. For patients with HF, left ventricular ejection fraction was obtained from formal transthoracic echocardiography performed during hospitalization and interpreted according to routine clinical practice by the treating cardiology team. No independent adjudication committee was used.

### Clinical data collection and AI evaluation

After completion of clinical care, physicians who had not participated in patient management retrospectively extracted demographic, clinical, laboratory, electrocardiographic, imaging, treatment, and outcome data using a standardized case report form. Relevant patient information was subsequently entered into four independent AI systems: ChatGPT (GPT-5.5 Instant; OpenAI), ChatGPT Plus (GPT-5.5 Instant; OpenAI), DeepSeek (DeepSeek-V3-0324; DeepSeek AI), and an ESC guideline-based RAG system. All evaluations were performed between 20 and 28 May 2026, using the publicly available web interfaces and the default models available at that time, without manual model selection or parameter modification. The AI systems were therefore not applied contemporaneously during the July–November 2025 patient-enrolment period; all cases were evaluated retrospectively within the same 8-day evaluation window. No model, prompting strategy, or study pipeline configuration was intentionally changed during this period. Each patient was evaluated in a new independent conversation for every AI system. No previous patient cases, conversation history, or responses from other models were available during evaluation. All models received identical information and answered the same predefined questionnaire. The systems were selected *a priori* to represent widely accessible contemporary general-purpose AI platforms commonly available to clinicians and the public, while also allowing comparison with a domain-specific, ESC guideline-grounded RAG approach. ChatGPT and ChatGPT Plus were included to reflect commonly used standard and subscription-based access pathways within the same commercial ecosystem, DeepSeek was included as an independently developed general-purpose model from a different provider, and the RAG system was included to evaluate whether explicit guideline retrieval altered clinical reasoning and recommendation patterns. The study was not designed to provide an exhaustive comparison of all available AI platforms.

The questionnaire consisted of 12 predefined questions. Questions 1–5 evaluated the initial diagnostic assessment (differential diagnosis, additional investigations, admission decisions, and prognosis) using only the information available at presentation, without disclosure of the final diagnosis or echocardiographic findings. Immediately after completion of Question 5, the diagnosis of HF (HFrEF, HFmrEF, or HFpEF, as applicable) together with the echocardiographic findings was disclosed to the AI models, after which they answered the remaining questions regarding prognosis, treatment, timing of GDMT, follow-up investigations, and estimated hospitalization duration.

For patients in the non-HF comparator cohort, only the pre-diagnosis section of the questionnaire (Questions 1–5) was completed, and no diagnostic information was subsequently disclosed to the AI models.

AI responses were independently reviewed and recorded by physicians who had not participated in patient management. The physician comparator consisted of physicians from the cardiology service, including HF specialists, cardiology specialists and physicians in cardiology specialist training, who were responsible for routine management of the hospitalized patients. For therapeutic analyses, physician management decisions represented routine clinical care and served as the clinical reference comparator for agreement analyses rather than an independent gold standard of clinical correctness. The standardized AI input form and the complete questionnaire are provided in Supplementary Appendix 1.

The RAG system was developed as a structured, ESC-guideline-grounded clinical reasoning pipeline for retrospective analysis of HF admissions. It combined retrieval from indexed European Society of Cardiology Clinical Practice Guidelines with reranking, structured JSON generation, citation-control rules, schema validation, workbook code mapping, and prognosis-calibration guardrails. For each case, the system generated standardized responses to pre- and post-diagnosis clinical questions, explicitly distinguishing guideline-supported recommendations from patient-specific clinical interpretation, thereby enabling reproducible and auditable comparison with physician assessments and non-RAG language-model outputs.

Implementation and preprocessing of AI inputs. For the three publicly accessible comparison systems (ChatGPT, ChatGPT Plus, and DeepSeek), the standardized case prompts were entered through the publicly available web interfaces during the prespecified evaluation window described above. The ESC-guideline RAG system was implemented as a Python pipeline rather than as a web-interface workflow. Patient data were not entered as raw charts. After physician extraction into a standardized case-report form, variables were de-identified and converted into standardized case vignettes with fixed field order and wording. The pipeline separated the information available at emergency presentation from post-diagnosis information; final diagnosis and echocardiographic findings were withheld for Questions 1–5 and disclosed only for post-diagnosis questions in the HF cohort. No outcome data, physician decisions used as comparators, or responses from other AI systems were included in the prompts.

RAG integration. The ESC guideline-based RAG system combined retrieval of relevant ESC Clinical Practice Guideline recommendations with LLM reasoning before response generation. Retrieved guideline passages were incorporated into the standardized patient prompt, allowing recommendations to be grounded in contemporary ESC guidance while preserving the same questionnaire and patient information provided to the comparator AI systems. A detailed technical description of the RAG implementation is provided in the Supplementary Methods.

### Study outcomes

The primary objective was to compare AI-generated recommendations with physician decision-making across the diagnostic and therapeutic management of patients with AHF.

Diagnostic performance was evaluated using the balanced diagnostic cohort comprising 100 patients with acute HFrEF and 100 patients with non-HF causes of acute dyspnoea. Diagnostic accuracy was assessed for (i) identification of HF as the primary diagnosis and (ii) inclusion of HF among the three most likely differential diagnoses. The latter analysis reflects the clinically relevant emergency department scenario in which HF is considered within the initial differential diagnosis rather than necessarily ranked first.

For evaluation of the primary diagnosis, HFrEF, HFmrEF, HFpEF, acute decompensated HF, and combined respiratory infection with HF were considered correct HF diagnoses. For the top-three analysis, HF was considered correctly recognized if any of these diagnoses appeared among the three proposed differential diagnoses.

Secondary outcomes included recommendations regarding emergency department investigations, initial treatment during the acute presentation, initiation of GDMT during hospitalization after confirmation of the HF diagnosis, timing of treatment initiation, prognosis, hospitalization duration, and prediction of intensive care admission, intubation, and in-hospital mortality.

For intubation analyses, physicians classified the need for invasive mechanical ventilation as either yes or no, whereas AI models could additionally respond possible. Because this response indicated recognition of a potential need for invasive ventilation during the patient’s clinical course, possible and yes responses were combined for the primary agreement analysis, resulting in a binary outcome (intubation indicated vs. not indicated).

### Statistical analysis

Continuous variables are presented as mean ± standard deviation or median (interquartile range), as appropriate, while categorical variables are presented as frequencies and percentages. Agreement between physician and AI recommendations was evaluated using Cohen’s kappa coefficient (κ), and systematic differences in paired categorical responses were assessed using McNemar’s test. Differences in paired binary diagnostic performance among the four AI models were evaluated using Cochran’s *Q* test. Pairwise comparisons between AI models were subsequently performed using McNemar’s test when the overall Cochran’s *Q* test was statistically significant. Diagnostic performance was assessed by calculating sensitivity, specificity, positive predictive value (PPV), negative predictive value (NPV), and accuracy, each with corresponding 95% confidence intervals (CIs). Because the diagnostic cohort was deliberately balanced to include equal numbers of patients with and without HF, PPV and NPV reflect the 50% disease prevalence of the study sample and should be interpreted as sample-specific measures rather than estimates applicable to an unselected emergency-department population. Differences in ordinal outcomes, including timing of GDMT and prognosis, were evaluated using the Wilcoxon signed-rank test. Sensitivity and specificity were calculated for prediction of critical clinical outcomes and for HF diagnostic performance. Separate multivariable logistic regression analyses were performed for physicians and each AI model to identify clinical predictors of ACEi/ARB/ARNI, β-blocker, SGLT2 inhibitor, and mineralocorticoid receptor antagonist recommendations. Predictor variables included systolic and diastolic blood pressure, serum urea, creatinine, sodium, potassium, left ventricular ejection fraction, age, and heart rate. Results are reported as odds ratios (ORs) with 95% CIs. Logistic regression model performance was assessed using the omnibus likelihood ratio test, Nagelkerke’s *R*^2^, and the Hosmer–Lemeshow goodness-of-fit test. Logistic regression models that failed to converge because of quasi-complete separation were not interpreted. All statistical analyses were performed using IBM SPSS Statistics, version 30 (IBM Corp., Armonk, NY, USA), and MedCalc (MedCalc Software Ltd., Ostend, Belgium) for calculation of diagnostic performance measures and their 95% CIs. A two-sided *P*-value of <0.05 was considered statistically significant.

### Ethics approval

The study protocol was reviewed and approved by the Scientific Committee of Sotiria General Hospital, Athens, Greece (Protocol No. 15202, approved on 12 June 2025). The study was conducted in accordance with the principles of the Declaration of Helsinki. This was a retrospective observational study, and AI recommendations were generated retrospectively without influencing patient management.

## Results

### Baseline characteristics

The study included 200 consecutive patients presenting with acute dyspnoea, comprising 100 patients with acute HFrEF and 100 patients with pulmonary diseases serving as a disease-negative comparator group. Baseline demographic and clinical characteristics of both groups are summarized in *Table 1*.

**Table 1:** Baseline characteristics of the study population

Baseline comparison between study groups			
Characteristic	Pulmonary disease (*n* = 100)	Acute HFrEF (*n* = 100)	*P*-value
Age, years	71.9 ± 9.5	74.5 ± 12.4	0.09
Male sex, *n* (%)	71 (71.0)	72 (72.0)	0.88
Systolic blood pressure (mmHg)	133.6 ± 21.6	129.8 ± 25.3	0.26
Diastolic blood pressure (mmHg)	71.8 ± 11.5	79.2 ± 16.2	<0.001
Heart rate (beats/min)	88.0 ± 22.3	88.5 ± 34.6	0.90

Characteristics of the acute HF cohort	Value
Cardiac rhythm, *n* (%)	
Sinus rhythm	51 (51.0)
Atrial fibrillation	32 (32.0)
Other rhythms^a^	17 (17.0)
Left ventricular ejection fraction (%)	37.0 ± 5.7
Key laboratory findings	
White blood cells (×10^9^/L)	9.95 ± 3.35
Haemoglobin (g/dL)	14.2 ± 2.9
Platelets (×10^9^/L)	187.8 ± 47.7
BNP (pg/mL)	274 (133.5–1311.5) (*n* = 37)
Troponin I (ng/L)	465 (265.5–2368)
C-reactive protein (mg/dL)	1.69 (0.49–3.42)
Urea (mg/dL)	44.6 ± 9.7
Creatinine (mg/dL)	1.08 ± 0.36
Sodium (mmol/L)	137.6 ± 6.5
Potassium (mmol/L)	5.10 ± 1.18
Glucose (mg/dL)	184.4 ± 75.9

Data are presented as mean ± standard deviation, median (interquartile range), or *n* (%), as appropriate. Continuous variables in the baseline comparison were compared using independent-samples *t*-tests and categorical variables using Pearson’s χ^2^ test. Sample size is indicated where fewer than 100 observations were available.

BNP, B-type natriuretic peptide; BP, blood pressure; CRP, C-reactive protein; HF, heart failure; LVEF, left ventricular ejection fraction.

^a^Other rhythms include atrial flutter, paced rhythm, ventricular tachycardia, and high-grade atrioventricular block.

*Table 1* The groups were comparable with respect to age, sex distribution, systolic blood pressure, and heart rate, whereas patients with acute HFrEF had significantly higher diastolic blood pressure than those with pulmonary disease (*P* < 0.001).

Within the HFrEF cohort, patients demonstrated advanced systolic dysfunction with substantial comorbidity burden, consistent with a real-world population requiring optimization of GDMT. Detailed laboratory, electrocardiographic, and echocardiographic characteristics of the HFrEF cohort are presented in *Table 1*, while additional presenting symptoms, medical history, comorbidities, and extended laboratory and blood gas findings are provided in Supplementary material online, *Tables S1*–*S3*.

### Diagnostic performance

Diagnostic performance was evaluated in a deliberately balanced cohort comprising 100 patients with acute HFrEF and 100 patients presenting with alternative causes of acute dyspnoea (*Table 2*). When considering HF as the primary diagnosis, overall diagnostic performance differed significantly among the four AI systems (Cochran’s *Q* = 8.477, *P* = 0.037). ChatGPT Plus demonstrated the highest overall performance, achieving a sensitivity of 87.0% (95% CI 78.8–92.9), specificity of 86.0% (95% CI 77.6–92.1), accuracy of 86.5% (95% CI 81.0–90.9), and the strongest agreement with the reference diagnosis (κ = 0.730). ChatGPT achieved the second-highest sensitivity (84.0%; 95% CI 75.3–90.6) but lower specificity (70.0%; 95% CI 60.0–78.8), whereas ChatGPT RAG demonstrated balanced sensitivity of 77.0% (95% CI 67.5–84.8) and specificity of 78.0% (95% CI 68.6–85.7), with an overall accuracy of 77.5% (95% CI 71.1–83.1) and κ = 0.550. DeepSeek showed the lowest diagnostic performance, with a sensitivity of 74.0% (95% CI 64.3–82.3), specificity of 64.0% (95% CI 53.8–73.4), accuracy of 69.0% (95% CI 62.1–75.3), and κ = 0.380. Pairwise McNemar analyses identified nominal differences between ChatGPT and ChatGPT Plus (*P* = 0.03) and between ChatGPT and ChatGPT RAG (*P* = 0.01).

**Table 2:** Diagnostic performance of the evaluated AI systems for identification of heart failure in patients presenting with acute dyspnoea (*n* = 200)

(*A*) Heart failure identified as the primary diagnosis						
Model	Sensitivity, % (95% CI)	Specificity, % (95% CI)	PPV, % (95% CI)^a^	NPV, % (95% CI)^a^	Accuracy, % (95% CI)	Cohen’s κ
ChatGPT	84.0 (75.3–90.6)	70.0 (60.0–78.8)	73.7 (67.2–79.3)	81.4 (73.3–87.5)	77.0 (70.5–82.6)	0.540
DeepSeek	74.0 (64.3–82.3)	64.0 (53.8–73.4)	67.3 (60.7–73.2)	71.1 (63.2–78.0)	69.0 (62.1–75.3)	0.380
ChatGPT xPlus	87.0 (78.8–92.9)	86.0 (77.6–92.1)	86.1 (79.2–91.0)	86.9 (79.8–91.7)	86.5 (81.0–90.9)	0.730
ChatGPT RAG	77.0 (67.5–84.8)	78.0 (68.6–85.7)	77.8 (70.4–83.7)	77.2 (70.0–83.1)	77.5 (71.1–83.1)	0.550
Overall comparison: Cochran’s *Q* = 8.477, *P* = 0.037						
Pairwise comparison of overall diagnostic classifications (McNemar test; unadjusted)						

Comparison	*P*-value
ChatGPT vs. DeepSeek	0.627
ChatGPT vs. ChatGPT Plus	0.029
ChatGPT vs. ChatGPT RAG	0.011
DeepSeek vs. ChatGPT Plus	0.243
DeepSeek vs. ChatGPT RAG	0.108
ChatGPT Plus vs. ChatGPT RAG	0.860

(*B*) Heart failure included among the top three differential diagnoses		
Model	Sensitivity, % (95% CI)	Specificity, % (95% CI)
ChatGPT	96.0 (90.1–98.9)	15.0 (8.7–23.5)
DeepSeek	90.0 (82.4–95.1)	19.0 (11.8–28.1)
ChatGPT Plus	97.0 (91.5–99.4)	70.0 (60.0–78.8)
ChatGPT RAG	92.0 (84.8–96.5)	68.0 (57.9–77.0)

Overall comparison: Cochran’s *Q* = 8.477, *P* = 0.037.

Data are presented as percentages with 95% confidence intervals (CIs). Overall differences among models were assessed using Cochran’s *Q* test. Pairwise comparisons were performed using McNemar’s test. Cohen’s κ represents agreement between each large language model and the reference diagnosis.

CI, confidence interval; PPV, positive predictive value; NPV, negative predictive value; κ, Cohen’s kappa coefficient; RAG, retrieval-augmented generation; LLM, large language model; HF, heart failure; HFrEF, heart failure with reduced ejection fraction.

^a^PPV and NPV were calculated within the deliberately balanced diagnostic cohort (100 acute HFrEF and 100 non-HF acute dyspnoea patients; HF prevalence 50%) and are therefore sample-specific. These values should not be generalized to populations with different heart-failure prevalence.

When the diagnostic criterion was expanded to include HF among the three most likely differential diagnoses, sensitivity was 96.0% (95% CI 90.1–98.9) for ChatGPT, 90.0% (95% CI 82.4–95.1) for DeepSeek, 97.0% (95% CI 91.5–99.4) for ChatGPT Plus, and 92.0% (95% CI 84.8–96.5) for ChatGPT RAG. Corresponding specificity values were 15.0% (95% CI 8.7–23.5), 19.0% (95% CI 11.8–28.1), 70.0% (95% CI 60.0–78.8), and 68.0% (95% CI 57.9–77.0), respectively. Overall, differences in diagnostic performance among the four AI systems remained statistically significant (Cochran’s *Q* = 8.477, *P* = 0.037). Within this broader diagnostic definition, ChatGPT Plus and ChatGPT RAG demonstrated the most favourable balance between sensitivity and specificity among the evaluated AI systems. The complete distribution of primary diagnoses assigned by each AI model in patients with confirmed acute HFrEF is presented in Supplementary material online, *Table S4*.

### Guideline-directed medical therapy recommendations

Agreement between physicians and the evaluated AI models for initiation of GDMT is presented in *Table 3*, Panel A. Overall agreement varied according to medication class and model architecture. Agreement was highest for β-blocker recommendations, reflecting the widespread physician consensus regarding their use, whereas greater variability was observed for SGLT2 inhibitor and mineralocorticoid receptor antagonist recommendations. Agreement patterns differed depending on whether raw concordance or agreement beyond chance was considered. DeepSeek demonstrated the highest Cohen’s κ for ACEi/ARB/ARNI initiation, whereas ChatGPT had the highest κ for SGLT2 inhibitor and MRA recommendations. ChatGPT RAG achieved the highest raw agreement for SGLT2 inhibitor recommendations.

**Table 3:** Guideline-directed medical therapy recommendations: agreement with physicians and multivariable determinants of treatment recommendations

(*A*) Agreement between emergency physicians and AI systems					
Therapy	Model	Overall agreement, *n* (%)	Cohen’s κ	κ *P* value	McNemar *P* value
ACEi/ARB/ARNI	ChatGPT	82 (82.0)	0.151	0.004	<0.001
	DeepSeek	80 (80.0)	0.398	<0.001	0.82
	ChatGPT+	81 (81.0)	0.128	0.04	<0.001
	ChatGPT RAG	79 (79.0)	0.087	0.25	<0.001
β-Blocker	ChatGPT	90 (90.0)	−0.018	0.75	0.02
	DeepSeek	79 (79.0)	0.116	0.20	0.08
	ChatGPT+	81 (81.0)	−0.008	0.90	0.64
	ChatGPT RAG	87 (87.0)	0.060	0.49	0.58
SGLT2 inhibitor	ChatGPT	81 (81.0)	0.320	<0.001	0.64
	DeepSeek	56 (56.0)	0.092	0.17	<0.001
	ChatGPT+	66 (66.0)	−0.022	0.80	0.009
	ChatGPT RAG	87 (87.0)	0.249	0.009	0.26
MRA	ChatGPT	77 (77.0)	0.260	<0.001	<0.001
	DeepSeek	64 (64.0)	0.179	0.07	0.24
	ChatGPT+	66 (66.0)	0.030	0.75	0.06
	ChatGPT RAG	74 (74.0)	0.151	0.03	<0.001

(*B*) Multivariable logistic regression analysis of treatment recommendations				
Therapy	Model	Omnibus *P*	Nagelkerke *R*^2^	Independent predictors (*P* < 0.05)
ACEi/ARB/ARNI	Physicians	<0.001	0.528	Urea (−), potassium (+)
	ChatGPT	0.02*	—	Model did not converge*
	DeepSeek	<0.001	0.456	Potassium (−), age (−)
	ChatGPT+	0.11	0.579	None
	ChatGPT RAG	0.06	0.471	LVEF (−)
β-Blocker	Physicians	0.16	0.295	Diastolic blood pressure (+)
	ChatGPT	—*	—*	Model did not converge^a^
	DeepSeek	0.001	0.409	None
	ChatGPT+	0.27	0.209	None
	ChatGPT RAG	0.02	0.496	LVEF (−)
SGLT2 inhibitor	Physicians	0.04	0.339	Age (−), heart rate (+)
	ChatGPT	0.009	0.329	LVEF (−)
	DeepSeek	0.16	0.174	Age (−)
	ChatGPT+	0.27	0.160	None
	ChatGPT RAG	0.003	0.572	LVEF (−), creatinine (−)
MRA	Physicians	<0.001	0.563	Creatinine (−), LVEF (−)
	ChatGPT	0.02	0.457	LVEF (−)
	DeepSeek	<0.001	0.358	Age (−), LVEF (−)
	ChatGPT+	0.17	0.213	LVEF (−)
	ChatGPT RAG	0.02	0.496	LVEF (−)

Overall physician recommendation rates were 80%, 91%, 88%, and 72% for ACEi/ARB/ARNI, β-blockers, SGLT2 inhibitors, and MRA, respectively. Data shown in (*B*) are from multivariable logistic regression analyses. Only independent predictors with *P* < 0.05 are listed. Complete regression outputs, including odds ratios, 95% confidence intervals, and all covariates, are provided in Supplementary material online, *Tables S5*–*S8*.

ACEi, angiotensin-converting enzyme inhibitor; ARB, angiotensin receptor blocker; ARNI, angiotensin receptor–neprilysin inhibitor; κ, Cohen’s kappa; LVEF, left ventricular ejection fraction; MRA, mineralocorticoid receptor antagonist; RAG, retrieval-augmented generation; SGLT2, sodium-glucose cotransporter-2.

^a^Although the omnibus test reached statistical significance for the ChatGPT ACEi/ARB/ARNI model, regression coefficients could not be interpreted because of quasi-complete separation. Similarly, the ChatGPT β-blocker model failed to converge because nearly all patients were classified as candidates for β-blocker therapy.

Despite generally favourable agreement rates, Cohen’s κ values indicated only fair-to-moderate agreement beyond chance for several medication classes, suggesting meaningful differences in treatment recommendations despite similar overall recommendation frequencies. Detailed agreement statistics, including McNemar analyses, are presented in *Table 3A*.

### Timing of treatment initiation

Differences between physicians and AI models were more pronounced regarding the recommended timing of treatment initiation (*Table 4*). ChatGPT RAG demonstrated the closest agreement with physician recommendations and showed no significant differences for any medication class. In contrast, ChatGPT+ consistently recommended later initiation of all four components of GDMT. ChatGPT and DeepSeek also recommended delayed initiation of SGLT2 inhibitors compared with physicians, while DeepSeek additionally suggested later initiation of mineralocorticoid receptor antagonists. These findings indicate that although AI models generally identified the appropriate therapies, important differences remained regarding the urgency of treatment implementation.

**Table 4:** Time to initiation of guideline-directed medical therapy recommended by emergency physicians and AI systems

Therapy	Emergency physicians median (IQR), days	ChatGPT median (IQR), days (*P* value)	DeepSeek median (IQR), days (*P* value)	ChatGPT Plus median (IQR), days (*P* value)	ChatGPT RAG median (IQR), days (*P* value)
ACEi/ARB/ARNI	1 (1–2)	2 (1–2) *(0.424)*	2 (1.75–3) *(0.092)*	2 (2–3) *(0.004)*	2 (2–2) *(0.786)*
β-Blocker	2 (1–3)	2 (1–2) *(0.760)*	2 (1–3) *(0.650)*	3 (2–3) *(0.003)*	2 (2–2) *(0.178)*
SGLT2 inhibitor	2 (1–2)	3 (2–3) *(0.002)*	3 (2–4) *(0.001)*	4 (3–5) *(<0.001)*	2 (2–2) *(0.770)*
MRA	2 (1–2)	2 (2–3) *(0.071)*	3 (2–3) *(0.012)*	4 (3–4) *(<0.001)*	2 (2–2) *(0.690)*

Data are presented as median (interquartile range). Italicized values in parentheses represent *P* values for comparisons between each AI system and emergency physicians, calculated using the Wilcoxon signed-rank test. A *P* value < 0.05 was considered statistically significant.

ACEi, angiotensin-converting enzyme inhibitor; ARB, angiotensin receptor blocker; ARNI, angiotensin receptor–neprilysin inhibitor; MRA, mineralocorticoid receptor antagonist; SGLT2, sodium-glucose cotransporter-2.

### Clinical variables associated with treatment recommendations

The principal clinical variables associated with treatment recommendations are summarized in *Table 3B*. Complete multivariable regression coefficients for ACEi/ARB/ARNI and β-blocker recommendations are presented in Supplementary material online, *Tables S5* and *S6*. Detailed results for SGLT2 inhibitor and mineralocorticoid receptor antagonist models are described in Supplementary material online, *Tables S7* and *S8*. Physicians incorporated multiple patient characteristics into therapeutic decision-making, including blood pressure, renal function, potassium concentration, heart rate, age, and left ventricular ejection fraction. Among the evaluated AI models, ChatGPT RAG demonstrated the most consistent pattern of clinically plausible associations, with lower LVEF associated with recommendations across medication classes and lower creatinine associated with SGLT2 inhibitor initiation. DeepSeek primarily incorporated younger age into its recommendations, whereas ChatGPT identified reduced left ventricular ejection fraction as the principal determinant of SGLT2 inhibitor and MRA recommendations. ChatGPT+ demonstrated limited evidence of structured multivariable decision-making, with left ventricular ejection fraction remaining the only independent predictor for MRA initiation. Logistic regression models for ChatGPT ACEi/ARB/ARNI and β-blocker recommendations could not be reliably interpreted because of quasi-complete separation resulting from near-universal treatment recommendations.

Exploratory analyses of recommendations for advanced cardiac investigations, including coronary angiography and cardiac magnetic resonance imaging, are presented in the Supplementary Results.

### Initial treatment recommendations

Agreement between physician- and AI-recommended initial treatment varied according to the intervention. For intravenous diuretic therapy, all AI models demonstrated statistically significant agreement with physician recommendations, with ChatGPT+ showing the highest agreement (κ = 0.354, *P* < 0.001), followed by ChatGPT (κ = 0.230, *P* < 0.001), whereas DeepSeek and ChatGPT RAG showed slight but significant agreement (both κ = 0.119, *P* = 0.004). For vasodilator therapy, ChatGPT demonstrated the highest agreement (κ = 0.245, *P* = 0.004), while agreement was lower for ChatGPT+ (κ = 0.147, *P* = 0.108), DeepSeek (κ = 0.103, *P* = 0.232), and ChatGPT RAG (κ = 0.007, *P* = 0.880). DeepSeek achieved the highest agreement for antibiotic recommendations (κ = 0.395, *P* < 0.001), followed by ChatGPT+ (κ = 0.268, *P* = 0.006), ChatGPT (κ = 0.262, *P* = 0.002), and ChatGPT RAG (κ = 0.119, *P* = 0.032). For inotropic therapy, ChatGPT showed the highest concordance with physician recommendations (κ = 0.451, *P* < 0.001), followed by DeepSeek (κ = 0.418, *P* < 0.001), ChatGPT+ (κ = 0.396, *P* < 0.001), and ChatGPT RAG (κ = 0.194, *P* < 0.001). Similarly, for non-invasive ventilation, ChatGPT demonstrated the highest agreement (κ = 0.315, *P* < 0.001), followed by ChatGPT+ (κ = 0.236, *P* = 0.002), whereas agreement was not statistically significant for ChatGPT RAG (κ = 0.120, *P* = 0.201) or DeepSeek (κ = 0.027, *P* = 0.710). For intubation recommendations, after combining ‘possible intubation’ with ‘yes’ responses for the primary analysis, ChatGPT RAG demonstrated the highest agreement (κ = 0.492, *P* < 0.001), followed by ChatGPT (κ = 0.250, *P* = 0.002), while DeepSeek (κ = 0.063, *P* = 0.191) and ChatGPT+ (κ = 0.046, *P* = 0.483) showed no significant agreement beyond chance. In the secondary descriptive analysis, ‘possible intubation’ was suggested in 11%, 29%, 16%, and 0% of patients by ChatGPT, DeepSeek, ChatGPT+, and ChatGPT RAG, respectively. Overall, AI models demonstrated greater consistency for core guideline-directed HF therapies than for supportive or escalation interventions.

### Prognostic assessment

The prognostic performance of the evaluated AI models is summarized in *Table 5*. ChatGPT demonstrated the closest agreement with observed clinical outcomes and was the only model that showed no significant difference between predicted and observed prognosis. In contrast, DeepSeek and ChatGPT+ consistently overestimated disease severity, whereas ChatGPT RAG showed a tendency towards predicting prolonged hospitalization. For prediction of critical outcomes, ChatGPT+ achieved the highest sensitivity, whereas ChatGPT demonstrated the most balanced overall performance by combining good sensitivity with the highest specificity. The complete distribution of predicted prognostic categories is presented in Supplementary material online, *Table S9*. Wilcoxon signed-rank analyses and critical outcome prediction results are described in the Supplementary Results. Overall model performance across the principal clinical domains is summarized in *Figure 1*.

**Figure 1:**
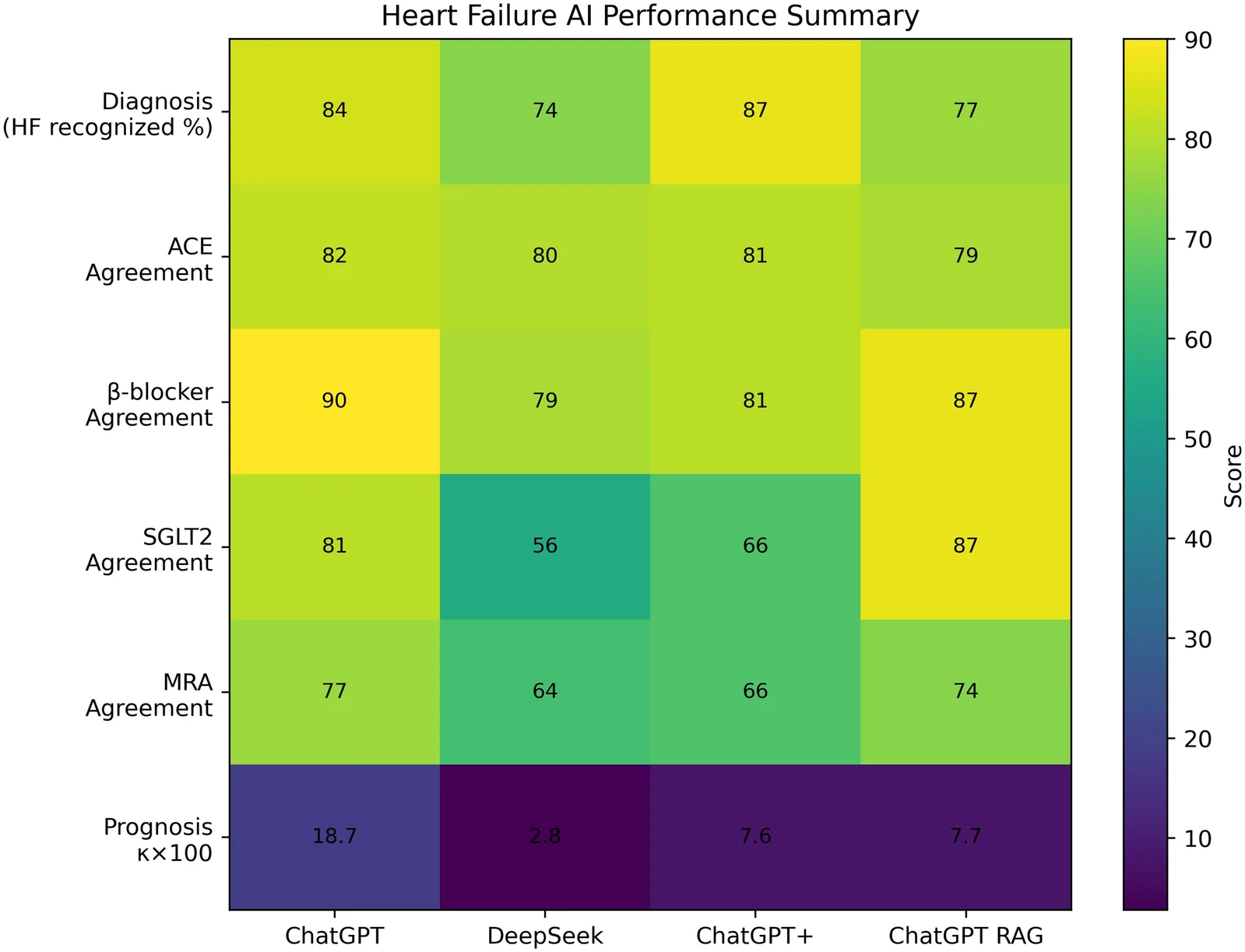
Overall performance of the evaluated AI models across major clinical domains. Heatmap summarizing model performance across the principal clinical tasks evaluated in this study, including heart failure diagnosis, guideline-directed medical therapy recommendations (ACE inhibitors/ARNIs, β-blockers, SGLT2 inhibitors, and mineralocorticoid receptor antagonists), and prognosis prediction. Numerical values represent the primary performance metric for each domain, while colour intensity reflects relative performance, with cooler colours indicating better performance. The figure illustrates complementary strengths among the evaluated AI systems. ChatGPT Plus demonstrated the highest overall diagnostic performance, ChatGPT RAG showed the closest alignment with physician-recommended treatment timing, and no single AI system consistently outperformed the others across all evaluated clinical domains.

**Table 5:** Prognostic performance of AI systems following heart failure diagnosis (*n* = 100)

Model	Predicted prognosis severity, median (IQR)	Cohen’s κ	Cohen’s κ (*P* value)	Wilcoxon *P* value	Critical outcome sensitivity (%)	Critical outcome specificity (%)	Overall prognostic tendency
ChatGPT	4 (4–5)	0.187	0.003	0.85	66.7	88.6	Closest agreement with observed outcomes
DeepSeek	5 (4–7)	0.028	0.57	<0.001	83.3	60.2	Frequently overestimated disease severity
ChatGPT+	6 (5–7)	0.076	0.07	<0.001	100.0	46.6	Highest sensitivity but substantial overestimation of severity
ChatGPT RAG	5 (4–6)	0.077	0.12	0.007	50.0	75.0	Predominantly predicted prolonged hospitalization

Critical outcome was defined as intensive care unit admission, endotracheal intubation, or in-hospital death. Prognostic severity was assessed using the predefined seven-category ordinal outcome scale. Wilcoxon signed-rank tests compared predicted and observed prognosis.

ICU, intensive care unit; IQR, interquartile range; κ, Cohen’s kappa.

## Discussion

The present study provides a comprehensive comparison of four contemporary AI systems across the diagnostic and therapeutic management of consecutive patients presenting with AHF. Unlike most previous studies that evaluated medical examination questions, isolated clinical scenarios, or single clinical tasks, our study assessed several clinically relevant stages of the pathway, including diagnosis, selection of investigations, initiation and timing of GDMT, and prognostic assessment. Several interesting findings emerged. First, using a balanced cohort comprising 100 patients with acute HFrEF and 100 patients with alternative causes of acute dyspnoea, all evaluated models demonstrated good diagnostic performance for HF, although substantial differences in discrimination were observed. ChatGPT Plus achieved the most favourable balance between diagnostic sensitivity and specificity, resulting in the highest overall diagnostic accuracy, whereas DeepSeek demonstrated the lowest overall performance. Second, when HF was considered among the three most likely differential diagnoses, sensitivity increased for all models, but this occurred at the expense of markedly reduced specificity for ChatGPT and DeepSeek. In contrast, ChatGPT Plus and the ESC guideline-based RAG model maintained high sensitivity while preserving substantially greater specificity, suggesting more efficient prioritization of clinically relevant differential diagnoses rather than indiscriminate expansion of diagnostic possibilities. Third, substantial variability remained in treatment recommendations and prognostic assessment, indicating that current AI systems are capable of supporting—but not replacing—physician decision-making.^8–10^ Importantly, these findings represent a time-specific snapshot of AI-system performance. Commercial AI models undergo frequent updates in model architecture, training, inference behaviour, and interface implementation; therefore, relative performance between systems may change over time, and the present results should be interpreted in the context of the model versions available during the May 2026 evaluation period.

These findings are consistent with the growing evidence that excellent performance on medical knowledge benchmarks does not necessarily translate into reliable real-world clinical reasoning. Hager *et al*. demonstrated that contemporary LLMs performed significantly worse than physicians when managing realistic clinical cases, showing important limitations in diagnostic performance, guideline adherence, laboratory interpretation, and robustness within clinical workflows.^8^ This observation is also supported by a randomized clinical trial by Goh *et al*., which found that although a standalone LLM performed strongly on diagnostic reasoning tasks, providing physicians with LLM assistance did not significantly improve diagnostic reasoning compared with conventional clinical resources, underscoring the complexity of integrating LLMs into real-world practice.^11^ Similar concerns have been highlighted in recent expert reviews, emphasizing that although LLMs have considerable potential as clinical decision-support tools, their safe implementation requires rigorous evaluation, disease-specific validation, and continued physician oversight.^9,10^ Our findings extend these observations to AHF, one of the most challenging cardiovascular syndromes encountered in emergency medicine. More recently, Brodeur *et al*. demonstrated that state-of-the-art reasoning-focused LLMs can achieve or exceed physician-level performance on challenging clinical reasoning tasks under experimental conditions, while emphasizing the need for prospective real-world validation before routine clinical implementation.^12^ Rather than demonstrating uniformly poor performance, however, our results indicate that diagnostic capability depends not only on overall model performance but also on the clinical endpoint being evaluated. While all models demonstrated very high sensitivity when HF was accepted among the three leading differential diagnoses, only ChatGPT Plus and the RAG model maintained satisfactory specificity, reflecting a more balanced diagnostic strategy. These findings suggest that contemporary LLMs differ not only in diagnostic accuracy but also in how they construct and prioritize differential diagnoses, highlighting important differences in clinical reasoning strategies that may influence their safe implementation in emergency cardiovascular care.^8^

Agreement was generally more consistent for core GDMT recommendations than for escalation interventions and prognosis, supporting the potential role of AI systems in promoting guideline-concordant care.^13^ Nevertheless, identifying the appropriate therapy proved easier than determining the optimal timing of treatment initiation. This distinction is clinically relevant because treatment timing in AHF requires integration of haemodynamic status, renal function, electrolyte abnormalities, and overall clinical stability, factors that extend beyond simple application of guideline recommendations.^9^ Among the evaluated models, the ESC guideline-based RAG system demonstrated the closest agreement with physicians regarding treatment timing and also showed the most consistent association between recommendations and clinically relevant variables such as left ventricular ejection fraction and renal function, suggesting a more clinically coherent pattern of treatment recommendations.^13^ Recent studies suggest that retrieval-augmented approaches improve evidence grounding and guideline adherence; however, their performance remains dependent on the clinical task and implementation strategy.^5,9,13^ Agreement was less consistent for acute supportive and escalation therapies, including vasodilators, non-invasive ventilation, and intubation, emphasizing that context-dependent bedside decisions were more difficult for the models than identification of standard long-term therapies.

An interesting observation was that no single model consistently outperformed the others. ChatGPT Plus demonstrated the most favourable balance between diagnostic sensitivity and specificity, achieving the highest overall diagnostic accuracy and agreement with the reference diagnosis, whereas ChatGPT demonstrated the most balanced prognostic performance and the RAG model showed the greatest consistency in treatment timing and physiologically plausible decision-making.^10^ Collectively, these findings suggest that the evaluated AI systems possess complementary rather than universal capabilities. Their greatest value may therefore lie in augmenting physician decision-making by improving diagnostic confidence, reinforcing adherence to evidence-based recommendations, and serving as a real-time cognitive support tool, rather than functioning as autonomous clinical decision-makers, a conclusion increasingly supported by recent evaluations of clinical LLM deployment.^5,8–10,13^ These findings support further investigation of how physician–AI collaboration might provide greater clinical value than unsupervised autonomous use.^11^

Prognostic assessment also proved challenging. ChatGPT demonstrated the closest overall agreement with observed clinical outcomes and was the only model without a significant difference between predicted and observed prognosis. In contrast, DeepSeek and ChatGPT+ tended to overestimate disease severity, with ChatGPT+ achieving the highest sensitivity for critical outcomes at the expense of substantially lower specificity. The RAG model more frequently predicted prolonged hospitalization. These findings suggest distinct patterns of prognostic miscalibration across models rather than a uniform tendency towards conservative risk estimation. If translated into practice, the observed tendency towards additional testing and overestimation of severity could increase resource utilization, although this was not directly evaluated in the present study.^8,10,14^ These findings further support the view that LLMs should currently function as clinical decision-support tools rather than autonomous decision-makers.^8,14,15^ Prospective evaluation of LLM-assisted workflows remains necessary to determine whether improvements in model performance translate into meaningful gains in patient outcomes and clinical efficiency.^12^

These observations suggest that future clinical AI systems should capitalize on the complementary strengths of different architectures rather than relying on a single foundation model. Hybrid systems integrating foundation models with disease-specific guideline retrieval and structured clinical knowledge may therefore represent the most promising direction for AI-assisted cardiovascular care.^7,9,13^

Beyond HF, AI is rapidly transforming cardiovascular medicine through applications including ECG interpretation, cardiac imaging, precision medicine, and clinical decision support.^14–17,20^ Increasing emphasis is also being placed on trustworthy, explainable, and multimodal AI to improve clinician confidence, transparency, and implementation in routine clinical practice.^8,9,17,18,20,21^ Explainable AI models have already demonstrated excellent performance in cardiovascular medicine while allowing clinicians to better understand the factors driving model predictions.^19,22^ Collectively, these developments support a future in which disease-specific LLMs, explainable AI, multimodal models, and guideline-based retrieval systems complement physician expertise while maintaining transparency, safety, and evidence-based care.^7,15,18,21^ Taken together, our findings suggest that future evaluations of clinical LLMs should extend beyond measures of diagnostic accuracy and agreement with clinicians to include assessment of reasoning consistency, guideline adherence, explainability, and clinical utility.

The present study has several strengths. It represents one of the first comparisons of multiple contemporary AI systems across several stages of the diagnostic and therapeutic pathway in consecutive patients with AHF. Unlike studies based exclusively on examination-style questions or simulated cases, our analysis used standardized retrospective case representations derived from patients managed in routine clinical practice, applied identical patient information across all AI systems, and assessed both agreement with physicians and the clinical variables associated with model recommendations.

Several limitations should also be acknowledged. This was a retrospective single-centre study, and the sample size was determined by the number of consecutive eligible patients available during the predefined study period rather than by an *a priori* power calculation, which may limit statistical precision and generalizability. In addition, the diagnostic analysis used a deliberately balanced cohort with equal numbers of acute HFrEF and non-HF acute dyspnoea cases; consequently, the reported PPV and NPV are specific to the 50% disease prevalence of this sample and should not be extrapolated to routine emergency-department populations, in which HF prevalence may differ substantially. Physician recommendations reflected routine clinical practice rather than an independently adjudicated gold standard and may themselves be subject to interobserver variability. Accordingly, higher physician–AI concordance should be interpreted as greater agreement with routine clinical decision-making rather than definitive evidence of greater clinical accuracy. Furthermore, the AI systems evaluated investigator-extracted structured clinical information rather than directly assessing patients and had no access to bedside examination, raw electrocardiograms, chest radiographs, echocardiographic images, or longitudinal follow-up. Thus, the present study evaluates clinical reasoning over standardized retrospective case representations rather than full bedside clinical performance. AI-system performance may change substantially over time as commercial models are updated; therefore, the present findings should be interpreted as a time-stamped assessment of the specific systems available during May 2026 rather than as permanent estimates of their relative performance. Prospective multicentre studies are therefore needed to determine whether AI-assisted clinical decision support improves patient outcomes, clinician efficiency, and adherence to evidence-based cardiovascular care.

## Conclusion

In this retrospective evaluation of consecutive real-world patients using standardized clinical case representations of consecutive patients presenting with AHF, contemporary AI systems demonstrated encouraging performance across multiple stages of clinical decision-making, particularly in syndrome recognition and recommendations for GDMT. However, clinically meaningful differences remained in exact HFrEF identification, treatment timing, and prognostic assessment, with no single model consistently outperforming the others. These findings indicate that the evaluated AI systems possess complementary strengths and are best viewed as clinical decision-support systems that augment, rather than replace, physician judgement. Future research should focus on prospective multicentre validation, integration of disease-specific knowledge, explainable and multimodal AI approaches, and retrieval-augmented systems to determine whether AI-assisted decision-making can safely improve patient outcomes, clinician efficiency, and adherence to evidence-based cardiovascular care.

## Lead author biography


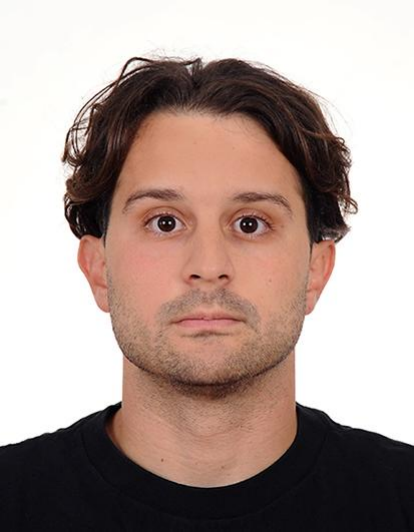


Georgios E. Zakynthinos, MD, is a cardiologist and PhD candidate at the University of Thessaly, Greece. His clinical and research interests include heart failure, cardiovascular critical care, heart–lung interactions, and artificial intelligence in cardiovascular medicine. He completed an Advanced Heart Failure and Mechanical Circulatory Support observership at the University of Utah. Dr Zakynthinos has authored more than 75 PubMed-indexed publications and serves as a reviewer for several international peer-reviewed journals in cardiology and critical care.

## Supplementary Material

ztag169_Supplementary_Data

## Data Availability

The data underlying this article are not publicly available because they contain potentially identifiable patient information. De-identified data may be made available by the corresponding author upon reasonable request, subject to institutional approval and applicable ethical and legal requirements.
