# Supplementary material for: Artificial intelligence versus physician decision-making in acute heart failure: a comparative evaluation of four contemporary AI systems
Source: Eur Heart J Digit Health. 2026 Oct 24;7(9):ztag169. doi: 10.1093/ehjdh/ztag169 (PMC13732570; doi:10.1093/ehjdh/ztag169)
Supplement: Supplementary file 1 [file ztag169_supplementary_data.docx]

***Supplementary Appendix 1. Standardized AI input form and predefined questionnaire***

The standardized form and questionnaire provided to each AI system are reproduced below. Questions 1–5 were answered before disclosure of the final diagnosis, whereas questions 6–12 were answered after the diagnosis of HFrEF and the echocardiographic findings were provided.

For the non-heart-failure comparator cohort, only Questions 1–5 were completed, and no diagnostic information was subsequently disclosed to the AI systems.

**A. Standardized patient information provided before diagnosis**

**Patient identification and demographic information**

| **Field** | **Entry / available option** |
| --- | --- |
| CODE |  |
| Age |  |
| Sex | Male - Female |

**Signs and symptoms**

Dyspnea on exertion – Dyspnea at rest – Orthopnea – Thoracic pain – Fever – Syncope – Tachycardia – Palpitations Hypotension

**Vital signs and initial clinical status**

| **Field** | **Entry / available option** |
| --- | --- |
| T | = o C |
| Blood pressure | = mmHg |
| Hemodynamically stable | Yes – No |
| Heart rate | = bpm |
| RR | = /min |
| Tachypneic |  |
| GCS | = /15 |
| SpO2 | = % |

**Laboratory exams**

| **Variable** | **Entry** | **Normal values** |
| --- | --- | --- |
| WBC | = Κ/μl | 4.6-10.20Κ/μl |
| Neutrophils | = Κ/μl | 1.79-7.50Κ/μl |
| HGB | = g/dl | 12.2-18.1g/dl |
| HCT | = % | 37.7-53.7% |
| PLT | = Κ/μl | 140.0-450.0Κ/μl |
| BNP | = pg/ml | <100pg/ml |
| TnI | = pg/ml | <34pg/ml |
| CRP | = mg/dl | <0.6mg/dl |
| Urea | = mg/dl | 15-45mg/dl |
| Creatinine | = mg/dl | 0.7-1.2mg/dl |
| Glucose | = mg/dl | 70-115mg/dl |
| Total bilirubin | = mg/dl | 0.3-1.2mg/dl |
| SGOT | = IU/L | 5-40IU/L |
| SGPT | = IU/L | 5-35IU/L |
| Γ-GT | = IU/L | 10-49IU/L |
| ALP | = IU/L | 25-125IU/L |
| Sodium | = mmol/l | 136-145mmol/L |
| Potassium | = mmol/l | 3.5-5.1mmol/L |
| APTT | = sec | 28.0-40.0 sec |
| PT | = sec | 12.0-15.0 sec |
| D-Dimers | = μg/ml | <0.5μg/ml |

**ABGs in fiO2**

| **Field** | **Entry** |
| --- | --- |
| pH | = |
| pO2 | = mmHg |
| pCO2 | = mmHg |
| Lac | = mmol/L |
| SpO2 | = % |

**ECG**

Sinus Rhythm, Atrial fibrillation - Atrial flutter - Hyperventricular arrhythmia - Ventricular tachycardia - BPM= /min – RBBB – LBBB - ST elevation – ST depression - Negative T - Biphasic T (IN aVL, I/ II,III,aVF/ V1-V2/ V3-V4/ V5-V6/ 6>one territory)

**CXR**

Increased cardiothoracic ratio - Blunting of the costophrenic angle (LEFT – RIGHT – BOTH SIDES) – Prominent interstitial markings (LEFT – RIGHT – BOTH SIDES) – Hyperinflation – Elevation of the hemidiaphragm (LEFT – RIGHT – BOTH SIDES) – Consolidation (LEFT – RIGHT – BOTH SIDES) – Cephalization – Alveolar infiltrates – Findings of sternotomy – Foreign body – Pacemaker – Dense opacities (LEFT – RIGHT – BOTH SIDES)

**Other exams**

**Patients’ history**

Heart failure with reduced ejection fraction - Heart failure with mildly reduced ejection fraction - Heart failure with preserved ejection fraction – Unspecified heart failure – Non ischemic HF – Coronary disease – Myocardial infarction – Coronary artery bypass graft – Percutaneous coronary intervention – Mild - Moderate - Severe - Unspecified Aortic Valve Stenosis – Mild - Moderate - Severe - Unspecified Mitral valve regurgitation - Permanent pacemaker - Paroxysmal atrial fibrillation – Permanent atrial fibrillation – Hypertension – Dyslipidemia – Diabetes mellitus II – Chronic kidney disease – Chronic obstructive pulmonary disease with home oxygen – Recent hospitalization for infection

**B. Questions for the AI before disclosure of the diagnosis**

**1.** Please list three potential diagnoses, in order of likelihood (from most to least probable).

**2.** Which further tests should be requested, and when should they be performed (Which day?, in the ER department or not?, after hospitalization or discharge?). Answer from most important to less important.

**3.** Should I discharge the patient?

**4.** If you suggest admission, in which department should the patient be admitted? (CARDIOLOGY DEPARTMENT, CICU, RESPIRATORY DEPARTMENT (PULMONOLOGISTS), INTERNAL MEDICINE, ICU, SURGICAL)

**5.** What is the prognosis for each diagnosis? Answer for each in a scale (one value, not a range):

**Prognostic scale used for Question 5**

| **Score** | **Label** | **Clinical Implication** |
| --- | --- | --- |
| 1 | Safe for Discharge from ER | Fully stable, no further inpatient management needed. |
| 2 | Short Observation (<24h) | Requires brief monitoring or diagnostic workup; likely to go home next day. |
| 3 | Short Admission (1–3 days) | Needs treatment/monitoring; can be discharged within 72h. |
| 4 | Standard Admission (3–7 days) | Requires therapy, imaging, and gradual stabilization; moderate severity. |
| 5 | Prolonged Admission (>7 days) | Complex management, likely complications, slow response to therapy. |
| 6 | High Risk of ICU | Hemodynamic instability; ICU likely. |
| 7 | High Risk of Intubation | Hemodynamic or respiratory instability; ICU likely. |
| 8 | High Risk of Death (In-hospital) | Critical illness, multiorgan risk, or very poor cardiac/respiratory reserve. |

**C. Information disclosed after completion of Questions 1–5**

Now I will tell you that the diagnosis was mainly acute HFrEF - HfmrEF – HfpEF. (There may be additional diagnoses that I will not provide—please take this into account in your workup.)

**Bedside ECHO**

LVEF= % - NORMAL SIZE LV - Dilated LV - RV normal size and contractility – RV dilated – decreased contractility mild – moderate – severe Mitral regurgitation – mild – moderate – severe Tricuspid regurgitation – mild – moderate – severe Aortic valve stenosis – mild – moderate – severe Aortic valve regurgitation - Pericardium - Inferior vena cava= mm with respiratory variation < - > 50%

**D. Questions for the AI after disclosure of the diagnosis**

**6.** What is the prognosis? Answer with the same scale as question 5 (one value, not a range).

**7.** What is the most likely cause of acute heart failure decompensation?

**8.** In which department should the patient be admitted? The CICU or the cardiology department?

**9.** What tests should I perform to identify the cause of heart failure? Present the answer as an algorithm. When there are two options for the same diagnostic purpose, choose the one you believe is most suitable.

**10.** What treatment plan do you recommend, and on which day should each component be initiated (try to be precise per day- answer separately for each day, do not use time periods. Do not make hypotheses or use 'if' statements—only provide definite answers based on the findings provided.)?

**11.** Which follow-up investigations and interventions are recommended during hospitalization and after discharge? Include preventive tests and interventions. Present the answer in order of importance and as a flowchart, where a positive result in one test may make subsequent tests unnecessary.

**12.** What is the estimated duration of hospitalization? Answer with the following scale (one value, not a range):

**Standardized Hospitalization Duration Scale (HDS-5)**

| **Score** | **Label** | **Definition** |
| --- | --- | --- |
| 1 | Discharged from ER | Patient is treated and discharged within the same day (<24h). |
| 2 | Short Stay (1–2 Days) | Brief observation or uncomplicated treatment course. |
| 3 | Standard Stay (3–5 Days) | Typical admission for acute decompensation or new diagnosis. |
| 4 | Prolonged Stay (6–10 Days) | Slow clinical response, need for additional evaluation or complex management. |
| 5 | Extended Stay (>10 Days) | Complicated hospitalization, often with ICU step-down or social issues. |

**E. Response recording fields**

| **AI system** | **Recorded response** |
| --- | --- |
| CHATGPT |  |
| CHATGPT+ |  |
| ChatGPT RAG system |  |
| DEEPSEEK |  |

***Supplementary Methods: Technical implementation of the ESC guideline-based RAG pipeline***

RAG integration. The RAG workflow was embedded at the prompt-generation stage. ESC Clinical Practice Guideline documents relevant to heart failure, acute coronary syndromes/myocardial infarction, pulmonary embolism, and cardiovascular diagnostic and treatment pathways were converted to text, divided into retrievable section/recommendation chunks, embedded, and stored in FAISS vector indices. For each patient and question set, the Python script generated fixed and case-triggered retrieval queries, retrieved candidate guideline chunks, reranked them using a cross-encoder, and supplied the highest-ranked excerpts to the LLM together with the standardized patient prompt. The model was instructed to return a structured JSON object containing the differential diagnosis, investigations, disposition, prognosis, treatment recommendations, guideline-support fields, and workbook codes. A validation layer checked schema completeness, code ranges, diagnosis-prognosis consistency, and citation rules. The RAG system therefore differed from the comparator LLMs by adding guideline retrieval, citation constraints, and deterministic post-processing/validation while keeping the patient case input and questionnaire structure aligned across systems.

***Supplementary Results***

# Symptoms, medical history, and initial clinical evaluation

The distribution of presenting symptoms is summarized in Supplementary Table S1. Dyspnoea was the predominant presenting symptom, followed by dyspnoea on exertion and paroxysmal nocturnal dyspnoea. Other symptoms, including chest pain, palpitations, dizziness, syncope, and fever, occurred less frequently.

Relevant cardiovascular and non-cardiovascular comorbidities are summarized in Supplementary Table S2. Coronary artery disease, hypertension, dyslipidaemia, atrial fibrillation, diabetes mellitus, and chronic obstructive pulmonary disease were the most common underlying conditions.

Additional laboratory and blood gas measurements that were not included in the main manuscript are presented in Supplementary Table S3.

# Detailed diagnostic distribution

The complete distribution of the primary diagnoses assigned by each AI model is presented in Supplementary Table S4. Although heart failure was recognized in most patients, the terminology used differed substantially among models. ChatGPT and DeepSeek most frequently identified acute decompensated heart failure, whereas ChatGPT plus (ChatGPT+) assigned HFrEF considerably more often. Alternative diagnoses, including NSTEMI, COPD exacerbation, pulmonary oedema, respiratory infection, atrial fibrillation, and valvular heart disease, were selected only occasionally.

# Detailed multivariable analyses of treatment recommendations

Complete multivariable logistic regression analyses are presented in Supplementary Tables S5–S8.

Supplementary Table S5 presents the regression analysis for ACEi/ARB/ARNI recommendations. Physician recommendations were independently associated with lower urea concentrations and higher potassium levels, whereas DeepSeek recommendations were associated with younger age and lower potassium. Lower LVEF independently predicted recommendations by ChatGPT RAG. The ChatGPT model could not be reliably interpreted because of quasi-complete separation.

Supplementary Table S6 presents the regression analysis for β-blocker recommendations. Higher diastolic blood pressure independently predicted physician recommendations, whereas lower LVEF was the only independent predictor identified for ChatGPT RAG. No independent predictors were identified for ChatGPT+, and the ChatGPT model failed to converge because of limited variability in recommendations.

Supplementary Table S7 summarizes the regression analysis for SGLT2 inhibitor recommendations. Physician recommendations were independently associated with younger age and higher heart rate, whereas ChatGPT recommendations were primarily influenced by lower LVEF. DeepSeek recommendations were associated with younger age, while ChatGPT RAG recommendations were independently associated with both lower LVEF and lower creatinine.

Supplementary Table S8 presents the regression analysis for mineralocorticoid receptor antagonist recommendations. Physician recommendations were independently associated with lower creatinine and lower LVEF. Lower LVEF also independently predicted recommendations by ChatGPT, ChatGPT+, and ChatGPT RAG, whereas DeepSeek recommendations were associated with both younger age and lower LVEF.

# Detailed prognostic distribution

The complete distribution of predicted prognostic categories compared with the observed clinical outcome is presented in Supplementary Table S9. ChatGPT produced a prognostic distribution that most closely resembled the observed outcomes, particularly for standard and prolonged hospitalization. DeepSeek and ChatGPT+ more frequently classified patients into severe prognostic categories, including ICU admission, intubation, and death, whereas ChatGPT RAG most commonly predicted prolonged hospitalization.

# Advanced cardiac investigation recommendations

**Coronary angiography recommendations.** Agreement between AI recommendations for coronary angiography and physician recommendations varied across models. ChatGPT RAG demonstrated the highest agreement (κ = 0.434, p < 0.001), followed by ChatGPT (κ = 0.310, p = 0.001), whereas DeepSeek (κ = 0.140, p = 0.068) and ChatGPT+ (κ = −0.034, p = 0.670) showed only slight or no agreement beyond chance. McNemar's test demonstrated no systematic difference between physician and ChatGPT RAG recommendations (p = 0.345), whereas significant differences were observed for ChatGPT (p = 0.041), DeepSeek (p < 0.001), and ChatGPT+ (p < 0.001). All four AI models recommended coronary angiography in every patient who ultimately underwent coronary revascularization (PCI or CABG), corresponding to 100% sensitivity in this subgroup.

**Cardiac magnetic resonance imaging recommendations.** Agreement between AI and physician recommendations for cardiac MRI was generally low. ChatGPT+ demonstrated the highest agreement (81%; κ = 0.207, p = 0.020), followed by DeepSeek (67%; κ = 0.156, p = 0.020) and ChatGPT (58%; κ = 0.131, p = 0.020). Although ChatGPT RAG achieved an overall agreement of 80%, agreement beyond chance was negligible (κ = −0.012, p = 0.899), reflecting its tendency to recommend MRI less frequently than physicians. McNemar's test showed significant systematic differences for ChatGPT (p < 0.001), DeepSeek (p < 0.001), and ChatGPT+ (p = 0.019), whereas no significant difference was observed for ChatGPT RAG (p = 0.263).

| **Supplementary Table S1.** Presenting symptoms of the study population (n = 100) | |
| --- | --- |
| **Presenting symptom** | **n (%)** |
| Dyspnea | 66 (66.0) |
| Dyspnea on exertion | 14 (14.0) |
| Paroxysmal nocturnal dyspnea | 9 (9.0) |
| Angina | 4 (4.0) |
| Non-ischemic chest pain | 7 (7.0) |
| Fever | 2 (2.0) |
| Syncope | 4 (4.0) |
| Dizziness | 6 (6.0) |
| Tachycardia | 3 (3.0) |
| Palpitations | 9 (9.0) |
| Hypotension | 3 (3.0) |
| Other presenting symptoms* | 40 (40.0) |
| *Includes symptoms that occurred infrequently and were therefore grouped into a single category. | |

| **Supplementary Table S2.** Past medical history and cardiovascular comorbidities of the study population (n = 100) | |
| --- | --- |
| **Medical history** | **n (%)** |
| **Heart failure history** |  |
| Previous HFrEF | 45 (45.0) |
| Previous HFmrEF | 10 (10.0) |
| Previous HFpEF | 0 (0.0) |
| Previous unspecified heart failure | 6 (6.0) |
| **Cardiovascular disease** |  |
| Aortic valve stenosis* | 10 (10.0) |
| Mitral regurgitation† | 9 (9.0) |
| Coronary artery disease‡ | 43 (43.0) |
| Atrial fibrillation§ | 42 (42.0) |
| Hypertension | 51 (51.0) |
| Implantable cardioverter-defibrillator | 18 (18.0) |
| Permanent pacemaker | 9 (9.0) |
| **Other comorbidities** |  |
| Chronic obstructive pulmonary disease | 27 (27.0) |
| Previous ischemic stroke (IAEE) | 10 (10.0) |
| Type 2 diabetes mellitus | 35 (35.0) |
| Dyslipidemia | 52 (52.0) |
| Chronic kidney disease | 17 (17.0) |
| Recent hospitalization | 5 (5.0) |
| *Aortic valve stenosis included 6 mild cases, 1 severe case, 1 unspecified severity, and 2 patients treated with transcatheter aortic valve implantation (TAVI).  †Mitral regurgitation included 5 mild and 4 severe cases.  ‡Coronary artery disease included 37 patients with previous percutaneous coronary intervention (PCI) and 6 with previous coronary artery bypass grafting (CABG).  §Atrial fibrillation included 21 paroxysmal and 21 permanent cases. | |
| **Abbreviations:** CABG, coronary artery bypass grafting; CKD, chronic kidney disease; COPD, chronic obstructive pulmonary disease; HFmrEF, heart failure with mildly reduced ejection fraction; HFpEF, heart failure with preserved ejection fraction; HFrEF, heart failure with reduced ejection fraction; ICD, implantable cardioverter-defibrillator; PCI, percutaneous coronary intervention; PPM, permanent pacemaker; TAVI, transcatheter aortic valve implantation. |  |

| **Supplementary Table S3.** Extended baseline laboratory and blood gas characteristics of the study population | |
| --- | --- |
| **Characteristic** | **Value** |
| Neutrophils (×10⁹/L) | 7.43 ± 3.07 |
| Hematocrit (%) | 44.0 ± 7.4 |
| Troponin I (3 h) (ng/L) | 1215 (372.5–3544.5) (n=23) |
| Total bilirubin (mg/dL) | 0.50 (0.45–2) (n=73) |
| AST/SGOT (U/L) | 49 (35.5–103) |
| ALT/SGPT (U/L) | 28 (19.5–47.5) |
| GGT (U/L) | 60 (40–232.5) (n=91) |
| ALP (U/L) | 97.6 ± 22.1 (n=85) |
| APTT (s) | 29 (27.5–34.5) (n=93) |
| PT (s) | 14 (13.5–16.5) (n=93) |
| D-dimer (μg/ml) | 1.07 (0.6–2.29) (n=93) |
| pH | 7.348 ± 0.073 (n=81) |
| pO₂ (mmHg) | 94.4 ± 36.0 (n=80) |
| pCO₂ (mmHg) | 38.8 ± 10.4 (n=80) |
| Lactate (mmol/L) | 2.57 ± 0.72 (n=81) |
| P/F ratio | 309.2 ± 198.1 (n=78) |
| **Data are presented as mean ± standard deviation or median (interquartile range), as appropriate. Sample size is indicated where fewer than 100 observations were available. Continuous variables are presented as mean ± standard deviation or median (Q1–Q3), where Q1 and Q3 represent the 25th and 75th percentiles, respectively.** | |
| **Abbreviations:** ALP, alkaline phosphatase; ALT, alanine aminotransferase; APTT, activated partial thromboplastin time; AST, aspartate aminotransferase; GGT, gamma-glutamyl transferase; P/F ratio, arterial oxygen partial pressure to inspired oxygen fraction ratio; PT, prothrombin time. | |

| **Supplementary Table S4.** Distribution of primary diagnoses assigned by each large language model in patients with confirmed acute HFrEF (n = 100) | | | | |
| --- | --- | --- | --- | --- |
| **First diagnosis** | **ChatGPT n (%)** | **DeepSeek n (%)** | **ChatGPT+ n (%)** | **ChatGPT RAG n (%)** |
| HFrEF | 17 (17.0) | 13 (13.0) | 61 (61.0) | 27 (27.0) |
| HFmrEF | 4 (4.0) | 3 (3.0) | 7 (7.0) | 2 (2.0) |
| HFpEF | 1 (1.0) | 0 (0.0) | 4 (4.0) | 1 (1.0) |
| Acute decompensated HF (unspecified) | 62 (62.0) | 56 (56.0) | 14 (14.0) | 44 (44.0) |
| NSTEMI | 7 (7.0) | 11 (11.0) | 7 (7.0) | 13 (13.0) |
| Acute pulmonary edema | 0 (0.0) | 2 (2.0) | 0 (0.0) | 3 (3.0) |
| Pulmonary embolism | 0 (0.0) | 1 (1.0) | 0 (0.0) | 0 (0.0) |
| COPD exacerbation | 1 (1.0) | 7 (7.0) | 0 (0.0) | 0 (0.0) |
| Respiratory infection | 1 (1.0) | 2 (2.0) | 0 (0.0) | 1 (1.0) |
| Respiratory infection with HF | 0 (0.0) | 2 (2.0) | 1 (1.0) | 3 (3.0) |
| Valvular heart disease | 1 (1.0) | 0 (0.0) | 0 (0.0) | 1 (1.0) |
| Ventricular tachycardia / NSVT | 0 (0.0) | 1 (1.0) | 1 (1.0) | 0 (0.0) |
| Atrial fibrillation | 3 (3.0) | 1 (1.0) | 3 (3.0) | 4 (4.0) |
| Other diagnoses | 3 (3.0) | 1 (1.0) | 2 (2.0) | 1 (1.0) |
| This supplementary table summarizes the distribution of primary diagnoses assigned by each large language model in the 100 patients with confirmed acute HF and is intended to characterize diagnostic preferences rather than diagnostic accuracy. Diagnostic performance metrics derived from the balanced cohort of 100 heart failure and 100 non-heart failure patients are presented in Table 2. | | | | |
| **Abbreviations:** HF, heart failure; HFrEF, heart failure with reduced ejection fraction; HFmrEF, heart failure with mildly reduced ejection fraction; HFpEF, heart failure with preserved ejection fraction; NSTEMI, non-ST-elevation myocardial infarction; COPD, chronic obstructive pulmonary disease; NSVT, non-sustained ventricular tachycardia. | | | | |

| **Supplementary Table S5.** Multivariable logistic regression identifying clinical factors associated with ACEi/ARB/ARNI prescription by physicians and AI models | | | | | |
| --- | --- | --- | --- | --- | --- |
| **Predictor** | **Physicians OR (95% CI)** | **ChatGPT** | **DeepSeek OR (95% CI)** | **ChatGPT+** | **RAG OR (95% CI)** |
| Systolic BP | 1.03 (0.99–1.08), p=0.198 | NS | 1.01 (0.98–1.05), p=0.580 | NS | 1.10 (0.96–1.25), p=0.163 |
| Diastolic BP | 1.08 (1.00–1.18), p=0.059 | NS | 1.06 (1.00–1.14), p=0.063 | NS | 1.00 (0.89–1.12), p=0.930 |
| Urea | **0.95 (0.90–1.00), p=0.030** | NS | 0.98 (0.95–1.02), p=0.401 | NS | 1.18 (0.95–1.47), p=0.144 |
| Creatinine | 1.88 (0.17–20.73), p=0.606 | NS | 0.46 (0.05–4.33), p=0.494 | NS | 0.001 (0.000–7.54), p=0.123 |
| Sodium | 1.02 (0.86–1.20), p=0.855 | NS | 1.03 (0.88–1.20), p=0.717 | NS | 1.27 (0.94–1.71), p=0.123 |
| Potassium | **4.84 (1.18–19.90), p=0.029** | NS | **0.28 (0.10–0.83), p=0.021** | NS | 0.20 (0.01–3.28), p=0.259 |
| LVEF | 0.98 (0.93–1.05), p=0.611 | NS | 1.02 (0.96–1.08), p=0.584 | NS | **0.83 (0.70–0.98), p=0.029** |
| Age | 0.97 (0.89–1.05), p=0.463 | NS | **0.91 (0.85–0.98), p=0.017** | NS | 0.93 (0.81–1.08), p=0.339 |
| Heart rate | 1.00 (0.97–1.02), p=0.712 | NS | 0.99 (0.97–1.01), p=0.384 | NS | 0.96 (0.92–1.01), p=0.131 |
| **NS = no statistically significant predictor (p>0.05).** | | | | | |

| **Supplementary Table S6.** Multivariable logistic regression analysis of clinical predictors associated with β-blocker recommendations | | | | | | | | | | |
| --- | --- | --- | --- | --- | --- | --- | --- | --- | --- | --- |
| **Predictor** | **Physicians OR (95% CI)** | **p** | **ChatGPT OR (95% CI)** | **p** | **DeepSeek OR (95% CI)** | **p** | **ChatGPT+ OR (95% CI)** | **p** | **ChatGPT RAG OR (95% CI)** | **p** |
| Systolic BP | 0.965 (0.920–1.011) | 0.134 | —* | — | 0.981 (0.947–1.016) | 0.282 | 1.004 (0.967–1.042) | 0.841 | 1.089 (0.961–1.235) | 0.181 |
| Diastolic BP | 1.116 (1.003–1.242) | 0.045 | —* | — | 1.073 (0.998–1.154) | 0.058 | 1.011 (0.944–1.083) | 0.746 | 1.005 (0.896–1.128) | 0.927 |
| Urea | 1.001 (0.954–1.051) | 0.970 | —* | — | 0.964 (0.926–1.004) | 0.078 | 0.972 (0.933–1.012) | 0.165 | 1.158 (0.960–1.396) | 0.125 |
| Creatinine | 0.231 (0.021–2.504) | 0.228 | —* | — | 0.779 (0.089–6.842) | 0.822 | 1.425 (0.194–10.440) | 0.728 | 0.000 (0.000–2.229) | 0.075 |
| Sodium | 0.876 (0.626–1.226) | 0.439 | —* | — | 0.977 (0.817–1.167) | 0.794 | 1.063 (0.907–1.245) | 0.452 | 1.288 (0.959–1.731) | 0.093 |
| Potassium | 0.724 (0.166–3.146) | 0.666 | —* | — | 1.095 (0.395–3.037) | 0.861 | 2.184 (0.745–6.405) | 0.155 | 0.250 (0.019–3.223) | 0.288 |
| LVEF | 1.000 (0.922–1.084) | 0.997 | —* | — | 1.002 (0.941–1.067) | 0.958 | 0.969 (0.907–1.035) | 0.350 | 0.829 (0.702–0.978) | 0.026 |
| Age | 1.005 (0.917–1.102) | 0.908 | —* | — | 0.962 (0.896–1.032) | 0.278 | 0.966 (0.896–1.040) | 0.356 | 0.943 (0.820–1.086) | 0.416 |
| Heart rate | 0.997 (0.972–1.023) | 0.831 | —* | — | 0.992 (0.973–1.011) | 0.400 | 1.005 (0.982–1.027) | 0.693 | 0.967 (0.924–1.013) | 0.155 |
| **Model χ² (p)** | **13.11 (0.158)** |  | **—*** |  | **27.65 (0.001)** |  | **11.15 (0.266)** |  | **19.53 (0.021)** |  |
| **Nagelkerke R²** | **0.295** |  | **—** |  | **0.409** |  | **0.209** |  | **0.496** |  |
| **Hosmer–Lemeshow p** | **0.578** |  | **—** |  | **0.958** |  | **0.462** |  | **0.996** |  |
| *The ChatGPT logistic regression model failed to converge because 93 of 94 patients were classified as candidates for β-blocker therapy, resulting in quasi-complete separation and unstable coefficient estimates. | | | | | | | | | | |
| **Abbreviations:** BP, blood pressure; CI, confidence interval; LVEF, left ventricular ejection fraction; OR, odds ratio. | | | | | | | | | | |

| **Supplementary Table S7.** Multivariable logistic regression analysis of clinical predictors associated with SGLT2 inhibitor recommendations | | | | | | | | | | |
| --- | --- | --- | --- | --- | --- | --- | --- | --- | --- | --- |
| **Predictor** | **Physicians OR (95% CI)** | **p** | **ChatGPT OR (95% CI)** | **p** | **DeepSeek OR (95% CI)** | **p** | **ChatGPT+ OR (95% CI)** | **p** | **ChatGPT RAG OR (95% CI)** | **p** |
| Systolic BP | 1.021 (0.977–1.068) | 0.359 | 1.004 (0.970–1.038) | 0.835 | 1.014 (0.989–1.041) | 0.275 | 1.016 (0.986–1.048) | 0.304 | 1.085 (0.963–1.222) | 0.182 |
| Diastolic BP | 0.997 (0.919–1.081) | 0.943 | 1.003 (0.946–1.064) | 0.922 | 0.987 (0.946–1.029) | 0.540 | 0.986 (0.939–1.035) | 0.564 | 1.009 (0.898–1.133) | 0.885 |
| Urea | 0.989 (0.947–1.034) | 0.631 | 0.976 (0.940–1.014) | 0.209 | 1.012 (0.984–1.040) | 0.417 | 0.990 (0.961–1.019) | 0.484 | 1.134 (0.968–1.328) | 0.119 |
| Creatinine | 0.625 (0.068–5.749) | 0.678 | 0.969 (0.145–6.490) | 0.974 | 0.294 (0.064–1.347) | 0.115 | 0.517 (0.110–2.427) | 0.403 | 0.000 (0.000–0.847) | 0.045 |
| Sodium | 1.072 (0.899–1.279) | 0.437 | 1.139 (0.988–1.314) | 0.074 | 0.984 (0.874–1.108) | 0.788 | 0.977 (0.849–1.124) | 0.740 | 1.298 (0.974–1.729) | 0.075 |
| Potassium | 1.496 (0.462–4.841) | 0.501 | 1.221 (0.469–3.178) | 0.682 | 0.609 (0.297–1.248) | 0.175 | 1.024 (0.481–2.179) | 0.951 | 0.279 (0.029–2.716) | 0.272 |
| LVEF | 0.992 (0.919–1.071) | 0.839 | 0.917 (0.860–0.978) | 0.008 | 0.996 (0.953–1.041) | 0.852 | 0.963 (0.917–1.012) | 0.134 | 0.830 (0.710–0.970) | 0.019 |
| Age | 0.909 (0.828–0.999) | 0.047 | 0.977 (0.919–1.038) | 0.448 | 0.957 (0.918–0.997) | 0.037 | 1.023 (0.980–1.069) | 0.293 | 0.954 (0.831–1.095) | 0.500 |
| Heart rate | 1.027 (1.000–1.054) | 0.050 | 1.018 (0.998–1.038) | 0.083 | 0.988 (0.975–1.002) | 0.091 | 1.005 (0.990–1.020) | 0.538 | 0.971 (0.928–1.016) | 0.198 |
| **Model χ² (p)** | **17.98 (0.035)** |  | **21.99 (0.009)** |  | **13.11 (0.158)** |  | **11.11 (0.268)** |  | **25.24 (0.003)** |  |
| **Nagelkerke R²** | **0.339** |  | **0.329** |  | **0.174** |  | **0.160** |  | **0.572** |  |
| **Hosmer–Lemeshow p** | **0.693** |  | **0.126** |  | **0.951** |  | **0.038** |  | **0.383** |  |
| **Abbreviations:** BP, blood pressure; LVEF, left ventricular ejection fraction; SGLT2, sodium glucose transporter; OR, odds ratio. | | | | | | | | | | |

| **Supplementary Table S8.** Multivariable logistic regression analysis of clinical predictors associated with MRA recommendations | | | | | | | | | | |
| --- | --- | --- | --- | --- | --- | --- | --- | --- | --- | --- |
| **Predictor** | **Physicians OR (95% CI)** | ***P*** | **ChatGPT OR (95% CI)** | ***P*** | **DeepSeek OR (95% CI)** | ***P*** | **ChatGPT+ OR (95% CI)** | ***P*** | **ChatGPT RAG OR (95% CI)** | ***P*** |
| Systolic BP | 0.984 (0.950–1.019) | 0.371 | 0.989 (0.936–1.046) | 0.709 | 1.000 (0.972–1.029) | 0.986 | 1.026 (0.988–1.067) | 0.183 | 1.089 (0.961–1.235) | 0.181 |
| Diastolic BP | 0.989 (0.932–1.049) | 0.706 | 1.022 (0.918–1.138) | 0.689 | 1.006 (0.958–1.056) | 0.812 | 0.995 (0.936–1.058) | 0.876 | 1.005 (0.896–1.128) | 0.927 |
| Urea | 0.992 (0.955–1.032) | 0.702 | 0.971 (0.905–1.042) | 0.409 | 0.989 (0.958–1.021) | 0.506 | 0.999 (0.964–1.036) | 0.966 | 1.158 (0.960–1.396) | 0.125 |
| Creatinine | **0.031 (0.002–0.480)** | **0.013** | 0.647 (0.034–12.422) | 0.773 | 0.494 (0.087–2.795) | 0.425 | 0.311 (0.049–1.992) | 0.218 | 0.000 (0.000–2.229) | 0.075 |
| Sodium | 1.090 (0.911–1.303) | 0.346 | 1.265 (0.974–1.641) | 0.078 | 0.919 (0.792–1.068) | 0.271 | 1.045 (0.892–1.224) | 0.585 | 1.288 (0.959–1.731) | 0.093 |
| Potassium | 1.247 (0.481–3.235) | 0.650 | 1.572 (0.229–10.789) | 0.646 | 1.171 (0.529–2.592) | 0.697 | 0.781 (0.307–1.988) | 0.604 | 0.250 (0.019–3.223) | 0.288 |
| LVEF | **0.857 (0.782–0.938)** | **<0.001** | **0.843 (0.736–0.965)** | **0.013** | **0.950 (0.902–1.000)** | **0.049** | **0.929 (0.873–0.989)** | **0.021** | **0.829 (0.702–0.978)** | **0.026** |
| Age | 0.992 (0.937–1.050) | 0.780 | 0.972 (0.855–1.105) | 0.663 | **0.925 (0.878–0.976)** | **0.004** | 1.016 (0.961–1.074) | 0.584 | 0.943 (0.820–1.086) | 0.416 |
| Heart rate | 1.011 (0.990–1.032) | 0.302 | 1.022 (0.985–1.061) | 0.252 | 0.993 (0.978–1.009) | 0.382 | 1.001 (0.982–1.021) | 0.889 | 0.967 (0.924–1.013) | 0.155 |
| **Model χ² (*P*)** | **46.50 (<0.001)** |  | **19.57 (0.021)** |  | **28.44 (<0.001)** |  | **12.82 (0.171)** |  | **19.53 (0.021)** |  |
| **Nagelkerke R²** | **0.563** |  | **0.457** |  | **0.358** |  | **0.213** |  | **0.496** |  |
| **Hosmer–Lemeshow *P*** | **0.369** |  | **0.967** |  | **0.880** |  | **0.755** |  | **0.996** |  |
| **Abbreviations:** BP, blood pressure; LVEF, left ventricular ejection fraction; MRA, mineralocorticoid receptor antagonist; OR, odds ratio. | | | | | | | | | | |

| **Supplementary Table S9.** Distribution of predicted prognosis following confirmation of HFrEF compared with the observed clinical outcome (n = 100) | | | | | |
| --- | --- | --- | --- | --- | --- |
| **Prognostic category** | **Actual outcome n (%)** | **ChatGPT n (%)** | **DeepSeek n (%)** | **ChatGPT+ n (%)** | **ChatGPT RAG n (%)** |
| Discharge from the emergency department | 1 (1.0) | 0 (0.0) | 0 (0.0) | 0 (0.0) | 0 (0.0) |
| Short admission (1–3 days) | 6 (6.0) | 3 (3.0) | 4 (4.0) | 0 (0.0) | 10 (10.0) |
| Standard admission (3–7 days) | 45 (45.0) | 49 (49.0) | 27 (27.0) | 24 (24.0) | 17 (17.0) |
| Prolonged admission (>7 days) | 36 (36.0) | 30 (30.0) | 24 (24.0) | 17 (17.0) | 45 (45.0) |
| ICU admission / High risk of ICU admission | 2 (2.0) | 11 (11.0) | 13 (13.0) | 32 (32.0) | 20 (20.0) |
| Intubation / High risk of intubation | 4 (4.0) | 5 (5.0) | 17 (17.0) | 16 (16.0) | 4 (4.0) |
| Death / High risk of death | 6 (6.0) | 2 (2.0) | 15 (15.0) | 11 (11.0) | 4 (4.0) |
| **Abbreviations:** HFrEF, heart failure with reduced ejection fraction; ICU, intensive care unit. | | | | | |
